# Microbiome-based therapeutics for Parkinson's disease

**DOI:** 10.1016/j.neurot.2024.e00462

**Published:** 2024-10-11

**Authors:** Adam M. Hamilton, Ian N. Krout, Alexandria C. White, Timothy R. Sampson

**Affiliations:** Department of Cell Biology, Emory University School of Medicine, Atlanta GA 30322, USA

**Keywords:** Microbiome, Parkinson's disease, Therapeutics, Gut-brain axis

## Abstract

Recent experimental and clinical data demonstrate a significant dysregulation of the gut microbiome in individuals with Parkinson's disease (PD). With an immense influence on all aspects of physiology, this dysregulation has potential to directly or indirectly contribute to disease pathology. Experimental models have bridged these associations toward defined contributions, identifying various microbiome-dependent impacts to PD pathology. These studies have laid the foundation for human translation, examining whether certain members of the microbiome and/or whole restoration of the gut microbiome community can provide therapeutic benefit for people living with PD. Here, we review recent and ongoing clinically-focused studies that use microbiome-targeted therapies to limit the severity and progression of PD. Fecal microbiome transplants, prebiotic interventions, and probiotic supplementation are each emerging as viable methodologies to augment the gut microbiome and potentially limit PD symptoms. While still early, the data in the field to date support continued cross-talk between experimental systems and human studies to identify key microbial factors that contribute to PD pathologies.

Throughout the life course, animals interact with a robust and diverse community of indigenous microorganisms at each environmentally exposed surface [1]. Anatomical sites including the gastrointestinal and genitourinary tracts, skin, and oral and nasal cavities, each harbor a unique composition of symbiotic microbiota [2]. Across human studies and experimental models, we now understand that these organisms are not merely commensal, but have demonstrated effects on numerous physiological systems of the host- including metabolic, immunologic, and neuronal pathways that can impact neurodegeneration [3]. While microbiome-dependent signaling is critical to health, dysregulation of these pathways can impact risk of disease, or even trigger pathologies. In turn, modulation of the microbiome, through microbiome transplants, enrichment with specific beneficial microbes, and dietary manipulations, can improve these signaling pathways, leading to beneficial effects on disease.

The systemic impacts of microbiome-derived signals can be observed as far reaching as the central nervous system (CNS). Over the last decades, emerging data have implicated the gut microbiome in the etiopathogenesis of many neurological disorders, most notably Parkinson's disease (PD) [4]. This review highlights the rationale for further exploration of microbiome contributions to PD, with a focus on the recent developments of various microbiome-based therapeutics (Fig. 1). Given a current lack of disease modifying therapies for PD, in conjunction with the tractability of the microbiome, these represent an important therapeutic pipeline.

**Fig. 1:**
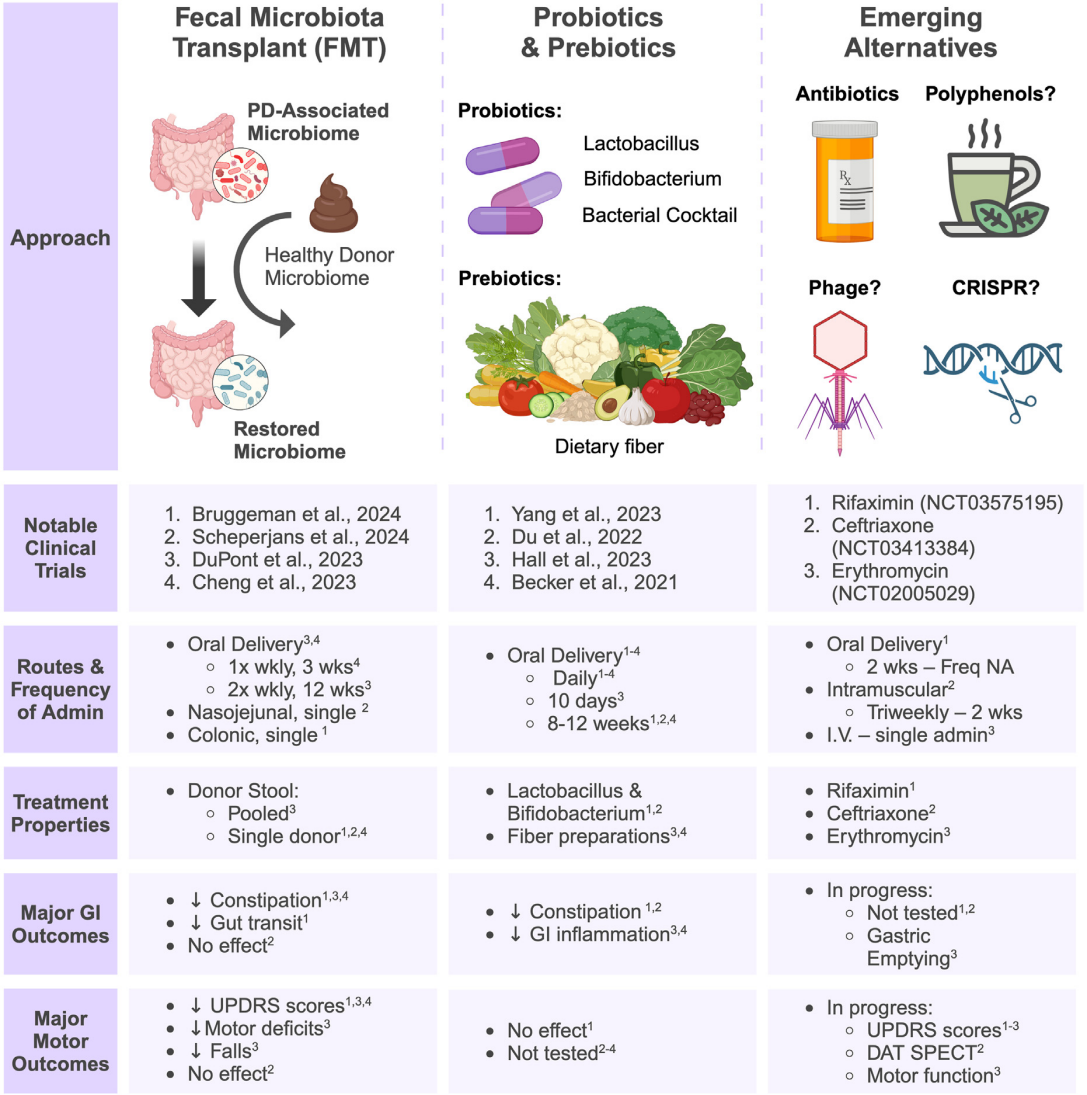
**Select microbiome-based therapeutics in recent and ongoing studies.** Selected comparisons of published and ongoing human studies to determine the safety and efficacy of microbiome-based therapies for individuals with Parkinson's disease.

## Microbiome Associations to Parkinson's Disease

It has long been observed that gastrointestinal (GI) dysfunctions, including inflammatory bowel disease (IBD) and intestinal dysmotility, often precede diagnosis of PD [5]. As far back as James Parkinson's original description of the Shaking Palsy, GI inflammation and other pathologies have been observed in PD [6]. Constipation is a significant risk factor for future PD diagnosis- ranging from 2 to 4 fold increased risk [[7], [8], [9]]. Individuals with IBD have up to a 32 ​% increased risk of PD, which can be mitigated with anti-TNF therapy [10,11], and appendectomy reduces risk by 19 ​% [12]. Based on a temporal spread of PD-associated synucleinopathy through connecting structures of the CNS, Braak and colleagues proposed that a microbial pathogen introduced at the olfactory epithelium or from the GI tract may be an initial insult to trigger a cascade of pathological events leading to neurodegeneration of vulnerable dopaminergic neurons of the substantia nigra [13,14]. These central events led to an investigation of the gut microbiome, as a potential contributor to intestinal inflammation and dysmotility, and/or a reservoir for an etiological pathogen.

Over the last decade, a number of studies have profiled the composition of the gut microbiome in individuals living with PD. Using both 16S and metagenomic sequencing, these studies have profiled stool and colonic mucosa microbiomes across a meta-cohort now greater than 1000 individuals that span across many geographic sites. Each study to date has observed differential abundance of a number of distinct intestinal bacteria. Formal meta-analyses of these studies have been performed across both 16S profiling and metagenomic analyses, revealing consistent and distinct PD-associated alterations to the gut microbiome [[15], [16], [17]]. Others have extensively reviewed the various taxa that are distinctly different between individuals with and without PD [4,18]. Generally, the PD-associated microbiome is characterized by increased *Akkermansia* sp., *Bifidobacterium* sp., *Enterobacteriacee*, and *Lactobacillus* sp., and decreased *Faecalibacterium* sp., *Lachnospiraceae* sp., and *Roseburia* sp [15,16].

To begin understanding how and when the microbiome may first shift in association with disease, longitudinal human observational studies are necessary. Given the protracted pre-diagnosed phase of PD, assessing the microbiome of individuals at high risk over time would allow a determination of which features arise first. The question of whether the microbiome composition begins to shift prior to PD diagnosis or during disease is essential in placing the microbiome in an etiological context. Some early data in individuals with REM Behavioral Disorder (RBD), who have high likelihood of a later PD diagnosis, indicates that a few PD-associated bacterial species are different in RBD compared to unaffected controls [[19], [20], [21]]. Adding to this is an observation that constipation, a known risk factor for PD, is associated with depletion of certain taxa that are also depleted in PD (with or without constipation), particularly fiber fermenting species [22,23]. This suggests that the microbiome may begin to shift during the pre-diagnosis period, prior to manifesting the robust alterations observed in those diagnosed with PD. However, these data are recent and while others have begun to link prodromal PD phenotypes to particular microbiome compositions [22], to our knowledge, no study to date has followed the microbiome of at-risk individuals, for instance with known genetic links (*e.g.* LRRK2, SNCA) or phenotypes (*e.g.* RBD, IBD) through conversion to PD diagnosis.

While longitudinal studies prior to diagnosis are more limited, in further support of this notion, a small number of studies have profiled the gut microbiome longitudinally after PD diagnosis [[24], [25], [26], [27]]. While still relatively short-time periods (1–2 year follow-up), these studies suggest that the PD-associated differences from healthy controls largely persist. For instance, short-chain fatty acid (SCFA) producing bacteria are generally depleted in the PD-associated microbiome, and this underrepresentation persists through one or two years [24,25,27]. Combining longitudinal microbiome analyses with the severity of disease progression within individuals could identify biomarkers of progression and/or reveal microbes that may be acting to contribute to disease severity. For instance, the abundance of *Prevotella* sp. [27], or *Bifidobacterium* sp. [28] have been observed to correlate with individuals who have more rapid progression of disease [27].

Newer data suggest the existence of two primary etiological forms of PD- a body-first subtype characterized by a loss of activity in the peripheral innervation to the cardiac and gastrointestinal systems; and a brain-first subtype without this early peripheral neuropathy [29,30]. These forms may impact the GI environment differently, resulting in different microbiome compositions, particularly in the early stages of disease. The body-first subtype may also represent a form that is etiologically triggered by a pathogenic event in the GI tract and periphery, as originally proposed by Braak and recently supported with further post-mortem pathological evidence [13,31]. If there is an etiological component within the microbiome it may be more apparent in individuals with aspects of a body-first phenotype. Future studies which stratify individuals into brain-first or body-first subtypes, or reassess existing studies using phenotypic metadata to separate subtypes may reveal stronger compositional differences within the microbiome based on these PD phenotypes.

Understanding the compositional differences is only part of the larger picture of the microbiome's potential contributions to disease. The significant compositional differences of the PD-associated microbiome observed across studies correspond with robust alterations to microbial genetic capacity. This includes a shift in genetic components involved in microbial carbon utilization (increased proteolytic vs. saccharolytic pathways), dysregulation of numerous neuroactive metabolites, and increased abundance of immune stimulating molecules [32]. In these human observations, both the compositional and functional differences within the PD-associated microbiome are solely associations. While it is tempting to speculate from these data alone that microbiome alterations have the potential to contribute to aspects of disease- for instance etiology or progression- the presence of a distinct microbiome does not necessitate a contribution to disease per se. The intestinal community structure may be altered as an epiphenomenon of disease- shifting in the face of a dysregulated intestinal environment (e.g. dysmotility, altered absorption, inflammation, etc.) However, these predicted functional differences allow tangible and testable hypotheses to be formed regarding contributions of the gut microbiome to various aspects of disease-relevant physiologies.

## Overview of Experimental Evidence for Microbiome Contributions to PD

Testing the functional contributions of the microbiome to PD etiopathogenesis harkens back conceptually to Koch's postulates, identifying pathogenic organisms sufficient to trigger disease. To date, such studies in PD have relied on various animal models that mirror different aspects of disease. Gnotobiotic manipulations across experimental animal models provide precise control over the presence or absence of particular species of interest within the microbiome. The ability to colonize experimental animals with single microbes or groups of selected microbes is one powerful approach to test the necessity and sufficiency of specific microbial interactions. These platforms also allow the reconstitution of a human-derived microbiome into an animal host. However, these studies may miss key inter-species interactions, or the reconstitution may not always successfully engraft critical species. Despite known limitations of the various animal models of PD that recapitulate some aspects of the human disease, manipulating the microbiome in a number of systems has provided foundational evidence supporting the notion of microbiome-dependent contributions to disease.

One very broad method to test the contribution of the microbiome is through derivation of germ-free animals, or to treat with broad-spectrum antibiotics. In these conditions of wholly missing or depleted microbiomes, mouse models of PD-relevant synuclein pathology, such as the Thy1-hSNCA model that over-expresses human wildtype SNCA, display markedly limited pathology [33]. This includes decreased synuclein accumulation, neuroinflammation, and motor impairments. In contrast, broad spectrum antibiotic treatment in mice overexpressing the highly pathogenic A53T synuclein allele revealed limited effects of antibiotic treatment in these same pathological measures [34]. In toxicant models, such as 6-OHDA, MPTP, and rotenone exposures which degenerate dopaminergic neurons, germ-free status or treatment with broad-spectrum antibiotics has been observed to protect mice from neurodegenerative outcomes [[35], [36], [37], [38], [39]].

These studies broadly provide evidence that an indigenous microbiome contributes to PD pathologies, but do not address whether the PD-associated microbiome itself is pathogenic and/or whether specific microbes contribute to disease. Selective antibiotic treatments have been used to begin to pinpoint organisms with capacity to modulate neurological outcomes, particularly in models of Alzheimer's disease and hyperactivity [40,41]. While selective antibiotic treatments have not yet been performed in models of PD, a number of studies have exposed or colonized models to PD-associated species or whole microbiomes to assess pathogenic or protective responses. Microbiomes derived from individuals living with PD were observed to exacerbate motor impairments in the Thy1-hSNCA mouse model, providing early evidence that specific members or the microbiome as a whole can contribute to aspects of PD [33]. This is similar in the MPTP mouse model, where exposure to microbiomes derived from individuals with PD exacerbated motor impairments and neuron loss [42]. Conversely, exposure to microbiomes derived from healthy humans [42], or healthy mice [43] significantly limits pathogenic outcomes following MPTP treatment. These data further support a contribution by the gut microbiome to the severity of PD pathologies.

Specific microbes have begun to be identified with both beneficial and pathogenic properties in various models of PD. Members of the bacterial family *Enterobacteriaceae* are generally enriched in the PD-associated microbiome [32,44,45]. One species, *Proteus mirabilis* results in increased sensitivity to MPTP in mice, suggesting that the carriage of this organism is sufficient to modulate the vulnerability of the dopamine system [46]. Increased vulnerability can be recapitulated with *Proteus* LPS exposure [46], and other models of PD are limited when lacking the microbial pattern recognition receptors TLR4 or TLR2 dependent immune signaling [[47], [48], [49]] suggesting a role for microbiome-immune interactions in facilitating PD pathogenesis. This is further supported by evidence demonstrating an exacerbation of PD-relevant pathologies in the A53T transgenic mouse model when exposed to dextran sodium sulfate to induce intestinal inflammation [50]. Intestinal infection with *Citrobacter rodentium* is also sufficient to drive frank neurodegeneration in the PINK1 knockout model of PD through an immune-mediated mechanism [51].

Another potential mechanism of microbiome-mediated impacts on PD pathologies includes the production of bacterial amyloidogenic molecules [52]. Bacteria which produce these proteins are often observed to be enriched in the PD microbiome [32,44,53]. With emerging evidence that alpha-synuclein has the capacity to propagate from peripheral sites into the CNS [[54], [55], [56]], it has been postulated that cross-seeding or mimicry by bacterial amyloids could trigger this spread [57]. An unbiased screen in *C. elegans* identified the curli amyloid from *E. coli* as having the ability to trigger alpha-synuclein accumulation [58]. Exposure to curli-producing *E. coli* was further observed to increase aSyn pathology in aged rats [59], and greatly exacerbated pathology and motor outcomes in Thy1-hSNCA mice [60]. Other potential contributing factors derived from the microbiome are beginning to emerge. For instance, microbiome-dependent production of the metabolite trimethylamine via metabolism of carnitine and choline is increased in individuals with PD [61], and increases dopamine neuron vulnerability in animal models [62]. Metabolism of levodopa is also modulated by the microbiome, with select microbes including *Bifidobacteria, Enterococcus*, and *Eggerthella* quickly converting therapeutic levodopa to dopamine or tyramine, which are unable to be readily absorbed in the intestinal tract [[63], [64], [65]]. Small molecules designed to inhibit these bacterial-specific enzymes are able to restore efficacy of levodopa in animal models [63].

In light of this experimental evidence that the microbiome can contribute to aspects of disease, further studies have begun exploring whether restoration of the microbiome may provide beneficial outcomes. Specific organisms, including *Lactobacillus plantarum*, *Clostridium butyricum*, and *Bacillus subtilis*, have been tested in toxicant induced or synuclein-based animal models [[66], [67], [68]] for their abilities to limit neurodegeneration and/or accumulation of alpha-synuclein. While the exact mechanism of neuroprotection or anti-amyloid activity is not wholly fleshed out in these studies, they serve to provide some early evidence that specific microbes may indeed be beneficial to limit PD-relevant pathologies.

Outside of experimental platforms, manipulation of the human gut microbiome is relatively straightforward and with limited risks. There is a clear line to translation into human studies. These emerging methodologies- fecal microbiome transplants, probiotic supplementation, prebiotic/dietary interventions, and other next-generation techniques provide new tangible and conceptual avenues for the treatment of PD.

## Fecal Microbiome Transplants

Minimally invasive and relatively inexpensive, fecal microbiome transplant (FMT) is an attractive approach to robustly modify the microbiome. FMT is the replacement of an existing gut microbiome with that of a healthy donor(s). This often involves the patient undergoing a bowel preparation and/or antibiotic pretreatment prior to receiving oral FMT capsules, a nasoduodenal/jejunal infusion, or a colonic administration of prepared donor fecal material. The objective is to create a large shift in the microbiome composition, to remove pathogenic species and/or enrich microbes with beneficial functions.

FMT has been successfully used for over a decade to treat recurrent and antibiotic-resistant *Clostridoides difficile* infection (CDI) in humans [69]. However, the use of FMT and follow-up data regarding long-term efficacy and safety remain limited [70]. In 2021, the NIH-funded FMT National Registry was established to track outcomes and standardize care as FMT therapies become more prevalent. The registry reported that out of over 200 CDI patients across 20 national FMT treatment sites in the U.S. between 2017 and 2019, 90 ​% of patients experienced total restoration of intestinal health 6 months post-FMT with very few adverse side effects [71]. To date, the FDA has approved two FMT therapies for prevention of CDI recurrence, Rebyota® and VOWST™, which each differ in their composition and administration [[72], [73], [74]]. While FMT is not yet formally recognized as a therapy outside of CDI, there is growing consensus that FMT may be suitable for treatment-resistant gastrointestinal disorders with strong microbiome contributions. For instance, inflammatory bowel diseases [[75], [76], [77]], and removing antibiotic-resistant *Enterobacteriaceae* urinary tract pathogens from the intestinal reservoir [78]. While generally consider a safe procedure, there have been a small percentage of serious adverse events after FMT therapy, including aspiration, intestinal perforation, sepsis, bacteremia, and even death. In a recent metanalysis, such serious adverse events occurred in only 0.65 ​% of 5099 patients across numerous FMT trials, supporting the low, but not absent, risk of the procedure [79]. Altogether, these findings reliably demonstrate the ability of a healthy donor microbial community to restore normal function to the local intestinal environment.

Outside of the gastrointestinal tract, evidence of successful FMT therapy for disorders with microbiome contributions is much more limited. For acute infections, such as CDI, the outcome of FMT is straightforward in removing infectious *C. difficile* and repopulating with diminished beneficial taxa. Candidate diseases differ in their causes (i.e., genetics, environmental factors) and durations (i.e., recurrent, chronic), and while the overall goal of FMT is similar, there is more limited understanding in which specific taxa may be detrimental or beneficial in a disease state. In neurodegenerative diseases, like PD, there are now strong associations between microbiome composition and human disease, as well as numerous animal studies indicating a contribution by the microbiome and specific microbes to pathology. Indeed, several studies have found that FMT can restore markers of striatal pathology and/or dopaminergic depletion in a variety of toxicant-induced PD rodent models [42,43,[80], [81], [82], [83]]. Given the relative ease and safety of FMT, as well as its ability to markedly impact microbiome composition as a whole, FMT represents both a potential therapeutic and a tool to study microbiome impacts to PD. A number of small case-reports and uncontrolled studies of FMT for PD have been reported [84,85]. At the time of this writing, there have only been four published, placebo-controlled studies of FMT intervention in PD [[86], [87], [88], [89]], and one currently registered (NCT04837313). While each of these four studies was placebo-controlled and participants were less than 7 years diagnosed with mild-to-moderated PD symptoms, the studies differed in FMT administration (route, frequency), and their choice of placebo composition (*e.g.* bowel clearance or starch pills). However, in all cases patients tolerated FMT treatment well for the duration of each study and minimal to no adverse effects were reported.

The first of these studies [86] used orally administered capsules containing lyophilized, previously-frozen donor microbes. Capsules were administered twice weekly for a duration of 12 weeks, without any pre-conditioning steps, and participants were followed for an additional 9 months post-FMT. While the cohort studied was small (8 FMT, 4 placebo), the primary endpoints were safety and whether the microbiome composition could be shifted by this methodology. Some participants reported mild-moderate upper GI symptoms, however overall adverse effects were minimal. Recipients of FMT displayed a shift in beta diversity of their stool microbiomes over the course of the study, with *Lactobacillaceae, Limnochordaceae,* and *Peptostreptococcaceae* families increased relative to placebo at 1 week post-FMT intervention. Additionally, some exploratory endpoints were investigated including self-reported, subjective assessments of constipation, sleep quality, sense of smell, and motor performance, as well as objective GI contractions (via SmartPill transit). Subjective measures of symptoms and GI contractility were improved in FMT recipients relative to placebo 1 month after initiating FMT therapy, however at later timepoints there was no significant difference between treatment groups. In this study, the composition of the donor material was not well described. Across the study 6 unique donors were used, and two samples from each donor were pooled. It is not clear if certain donors were more efficacious than others, or whether particular features from the donor's microbiome most strongly associated with outcomes. Further, given the tapering of both subjective and objective measures, it is possible that this form of FMT would need continuous administration to maintain the restructured community.

A second recent study also utilized oral capsule delivery of an FMT product [87]. Using a larger cohort (N ​= ​27), with FMT delivered once weekly for 3 weeks, the primary outcome measure was objective scoring by the comprehensive Universal Parkinson's Disease Rating Scale (UPDRS). This clinical measure was significantly improved in those receiving FMT at 9 weeks following intervention, compared to placebo controls. In addition, secondary outcomes in GI functions, including increased number of weekly bowel movements improved IBS symptom severity scale, were also found in the FMT group. Other measures of cognitive function, such as the standardized MMSE and MoCA scores were improved following FMT, but measures of depression and anxiety were not. Interestingly, FMT-treated patients were able to be divided into responders and non-responders according to improvements in their clinical scores. The resultant microbiome post-FMT of these individuals was significantly different, with responders having a microbiome more similar to their donor's, characterized by a greater number of select Firmicutes taxa, including species within *Eubacterium, Clostridia,* and *Roseburia* genera. This indicates a role for either specific microbes with the capacity to engraft into the community through this paradigm, or the presence of a pre-existing factor of the host (or microbiome) that would prevent successful engraftment. Nonetheless, it does show a further association with particular bacterial taxa that corresponded with improvement in this study's outcome measures and highlights potential biomarkers for therapeutic efficacy.

Nasojejunal administration, which was one of the earliest routes of FMT, has also been performed for PD. A recent study used a single nasojejunal administration and followed PD symptoms by the UPDRS as its primary outcome measure over one year post-treatment [88]. While both FMT and placebo groups showed improvements in UPDRS motor scoring, those receiving FMT had a significantly greater improvement over the course of the study. In addition, GI transit, measured through radiopaque pellet imaging, was improved post-FMT as well, albeit at earlier points within the study. Cognitive assessments were not different between the treatment groups, and self-reported measures of fatigue were increased among those receiving FMT. While the motor improvements in those receiving FMT were fairly striking particularly given a single administration, it is interesting to note that placebo-treated individuals also improved in many measures. In this study, both placebo and FMT groups received a standard bowel preparation to clear the bowel of its contents. It is tempting to speculate given the improvement in this placebo group in particular that bowel clearance was sufficient to beneficially shift the microbiome. However, microbiome composition of donors and recipients was not reported, so it is unclear whether there were particular bacterial taxa that associated more strongly with benefit.

Most recently, a randomized, double-blind, placebo-controlled trial [89] also used a single infusion of FMT product following a bowel preparation, but using an intra-colonic administration. Of note, this study selected individuals with a defined dysbiotic microbiome, representing the best predicted candidates for FMT therapy. The primary outcome measure of UPDRS was not significantly different from placebo controls at 6 months post-treatment. However, the placebo group displayed a significantly worsened levodopa equivalent daily dose (LEDD). As both experimental arms of the study were maintained on their existing medication regimens, this suggests (but does not prove) that the placebo group indeed worsened relative to those receiving FMT, but that symptoms scored by the UPDRS were masked by the increased levodopa intake. It is also possible that the shared bowel preparation between experimental arms also provided some benefit, as observed in the previously described study [88]. Nonetheless, it is exciting to consider that FMT allowed a decreased need for levodopa to maintain similar disease symptoms on this time scale of progression.

With only 4 placebo-controlled studies to date, there are a number of critical gaps that remain before therapeutic benefit (or lack thereof) may be more conclusively defined. Most importantly, in current published studies, the donor microbiomes and the engraftment of these donor microbes is not consistently described across studies. It is not known whether individuals who successfully showed the best engraftment also displayed the best primary outcomes. Or, whether across studies particular taxa were most closely associated with improved symptoms, irrespective of donor or route of administration. Studies to date have only described 6–12 months post-FMT, so the longevity of any physiological response to FMT in the context of PD is currently unclear. There is additionally no consistency in the route of FMT administration, nor the choice of placebo control. In the most recent studies, placebo groups also received a bowel preparation, which alone has the ability to shift the microbiome composition, at least in the short term. Beneficial outcomes relative to their pre-treatment baseline, in both placebo groups suggests bowel clearance alone may have a benefit greater than the expected psychophysiological placebo-effects. Only one study deliberately selected participants based on their microbiome composition [89]. Future work to identify those microbiome compositions that would be most likely to benefit (based on experimental models) is still needed. Meta-analysis across FMT studies of microbes that strongly associate with beneficial outcomes would reveal potential organisms to be deliberately selected for or against from donor and recipient to be used in more refined probiotic-type administrations.

## Emerging Probiotics in the Treatment of PD

In addition to the broad and efficacious restructuring of the microbiome associated with FMT, the more targeted therapeutic approach of administering probiotics, specific beneficial microbial species, has also gained significant interest in recent years. Probiotics can reliably modify the microbiome composition after microbial perturbation, yet generally have minimal impact on healthy subjects [90], making them a powerful and low risk tool for treatment of ailments with a contribution by the gut microbiome. A variety of probiotic therapeutics have been evaluated for their efficacy in treating aspects of PD, including GI dysfunction and motor impairment, in both humans and animal models. Data from numerous animal models has served as a substantial foundation for the study of probiotic benefit in PD. This includes diverse systems including 6-OHDA [91,92], MPTP [67,93,94], rotenone [68], and transgenic rodent models [95]. However, only a total of eleven published studies have investigated probiotic use in persons living with PD [[96], [97], [98], [99], [100], [101], [102], [103], [104], [105], [106]] and all have found favorable clinical outcomes with probiotic treatment (Table 1).

**Table 1:** Summary of outcomes across clinical studies of probiotics for Parkinson's disease.

Study		Probiotic treatment			Outcomes		
Study	Study Size (probiotic, control)	Probiotics used	Duration of treatment (weeks)	Prebiotics included?	Constipation	Motor	Other
Cassani et al. 2011	20, 20	*Lactobacillus casei* Shirota	5	No	Improved	Not tested	
Barichella et al. 2016	80, 40	*Streptococcus salivarius* subsp thermophilus, *Enterococcus faecium*, *L. rhamnosus* GG, *L. acidophilus*, *L. plantarum, L. paracasei, L. delbrueckii* subsp bulgaricus, *Bifidobacterium breve*, and *B. animalis* subsp lactis	4	Yes (fiber, incld fructooligosaccharides)	Improved	Not tested	
Georgescu et al. 2016	20, 20	*Lactobacillus acidophilus* and *Bifidobacterium infantis*	12	No	No change	Not tested	Improved abdominal pain and bloating
Borzabadi et al. 2018	25, 25	*Lactobacillus acidophilus, Bifidobacterium bifidum*, *L. reuteri*, and *L. fermentum*	12	No	Not tested	Not tested	Improved markers of inflammation and insulin metabolism
Tamtaji et al. 2019	30, 30	*Lactobacillus acidophilus*, *Bifidobacterium bifidum*, *L.reuteri*, and *L. fermentum*	12	No	Not tested	Improved	Decreased high-sensitivity C-reactive protein and improved insulin-related measures.
Ibrahim et al. 2020	22, 26	Multi-strain probiotic, Hexbio, which consists of *Lactobacillus acidophilus*, *L. casei, L. lactis, Bifidobacterium infantis*, and *B. longum*	8	Yes, fructooligosaccharides	Improved	No change	Found no changes in non-motor symptoms.
Tan,et al. 2021	34, 38	*Lactobacillus acidophilus, L. reuteri, L. gasseri, L. rhamnosus, Bifidobacterium bifidum, B. longum, Enterococcus faecalis*, and *E. faecium*	4	No	Improved	Not tested	No change in fecal calprotectin.
Sun et al. 2022	48, 34	*Bifidobacterium animalis* subsp. lactis Probio-M8	12	No	Improved	Improved	Improved sleep and anxiety measures. Changed microbiome composition. Increased serum acetate and dopamine.
Du et al. 2022	23, 23	*Bacillus licheniformis, Lactobacillus acidophilus, Bifidobacterium longum*, and *Enterococcus faecalis*	12	No	Improved	Not tested	Changed microbiome structure.
Yang et al. 2023	65, 63	*Lacticaseibacillus paracasei* strain Shirota	12	No	Improved	No change	Improved measures of non-motor symptoms. Increased levels of L-tyrosine. Changed microbiome structure.
Ghalandari et al. 2023	15, 15	Comflor® capsules containing L*actobacillus plantarum, Lactobacillus casei, Lactobacillus acidophilus, Lactobacillus bulgaricus, Bifidobacterium infantis, Bifidobacterium longum, Bifidobacterium breve*, and *Streptococcus thermophilus*	8	No	Improved	No change	

All eleven published studies to date used probiotic formulations consisting of *Lactobacillus* or *Bifidobacterium* species, or most often, a probiotic cocktail containing various species including multiple species from those genera. The choice of probiotic species is of particular interest as these genera are already found consistently elevated in the fecal microbiome of people living with PD [15,32]. It is unclear what would cause these organisms to be elevated in the PD-associated intestinal environment. Some of these organisms metabolize levodopa [[63], [64], [65]], and so may be enriched in response to PD treatment. As such, using these species as probiotics may also have direct effects that limit the efficacy of levodopa therapeutics. Certain *Lactobacillus* and *Bifidobacterium* species can promote beneficial immune responses, can limit growth of opportunistic pathogens, and improve intestinal integrity and mucous production [[107], [108], [109]]. However, whether the specific strains found in the PD-associated microbiome have these same benefits, or instead act in more unexpected and detrimental ways is currently unclear. Well-controlled study in gnotobiotic animal models would allow these complex facets to be differentiated. A small number of studies also combined the probiotic intervention with dietary supplementation- a method termed “synbiotics” [98,101]. Each of these provided or instructed an increase in dietary fiber intake, a key fermentable substrate for the microbiome, that may promote production of various beneficial metabolites.

The majority of these probiotic studies in humans with PD have focused on the improvement of GI dysfunction, most notably constipation, which affects 80–90 ​% of people with PD [5]. Given the roles of probiotic species present in fermented foods and their known benefits to GI health, including motility and permeability which are highly dysregulated in PD, it is a logical first step to investigate. Nine current studies had constipation as a primary outcome measure [[96], [97], [98], [99], [100], [101], [102], [103], [104]], and all but one [99] of those studies found that probiotic supplementation improved constipation, generally measured as frequency of bowel movements and stool consistency. The sole study that did not observe benefits on measures of bowel movements observed self-reported improvements in other GI symptoms including abdominal pain and bloating [99]. One multi-strain probiotic intervention also had significant improvement in quantitative measures of total GI transit time [101]. Collectively these studies indicate that probiotic interventions can have a substantial positive effect on constipation in PD.

A small number of studies have also begun to assess whether probiotic interventions can limit the hallmark motor and cognitive symptoms of PD. Of the five probiotic studies that assessed motor scores only two found significant improvements in UPDRS [100,106], while three found no significant differences [101,103,104]. However, there are caveats to both positive findings. One study reported that UPDRS-III motor scores improved with probiotic intervention relative to baseline levels (improvement from 19.38 to 17.45 on average) [100]. This is similar to some placebo outcomes in the previously described FMT trials, where placebo appeared to show significant benefit from baseline [88]. In this study, the placebo group received maltodextrin, a starch that can impact the microbiome [110] perhaps suggesting a benefit for this dietary fiber. The second beneficial study [106] noted an improved *combined* UPDRS score, which decreased by 4.8 (±12.5) points in the treatment group but increased by 3.8 (±13.0) points in the placebo group. As this score includes both motor and non-motor symptoms of PD, it is possible that improvement of non-motor symptoms lead to the significant difference between treatment groups rather than motor symptoms alone. Indeed, recent analyses suggest that combining non-motor and motor subscores across the UPDRS can result in inaccurate representations of the clinical state [111]. Three studies also measured mood-related non-motor symptoms [100,101,103], including anxiety and depression. Two of which found improvements associated with probiotic intervention [100,103] and one of which that found no differences between treatment groups [101]. Similar to effects on motor scores, one study noted improvements in both placebo and probiotic groups in measures of anxiety and sleep quality, relative to baseline measures, albeit with higher magnitudes of change in the probiotic treated group [100].

While symptom improvement has been the primary outcome measure, some studies assessed other pathological measures that are potentially acted on by the microbiome. These include inflammation, endocrine function, and relevant monoamine (*e.g.* dopamine) metabolism. Of three studies which investigated inflammation [96,105,106], two [105,106] found improvements to various inflammatory markers, including inflammatory gene expression in peripheral blood mononuclear cells, antioxidant capacity, and serum C-reactive protein. One, which measured levels of the immune marker fecal calprotectin, did not observe significant differences following probiotic treatment [96], perhaps suggesting probiotic effects are more robustly observed in circulation. Insulin signaling was also observed to be impacted by probiotic treatment in two studies that used an identical probiotic regimen. One observed upregulation of PPAR-γ [105], involved in insulin-signaling and adipogenesis. The other found that probiotics reduced total insulin levels and improved insulin resistance [106]. This may be critical for people with PD, since diabetes is highly co-morbid to PD. Two studies also observed interesting outcomes related to dopamine levels. One found that serum levels of dopamine where increased relative to placebo [100], and another found an increase in the dopamine precursor (and metabolite) L-tyrosine [103]. Since dopamine itself is not brain-penetrant and L-tyrosine levels may indicate a flux toward dopamine degradation, it is unclear whether these markers are wholly associating with a beneficial outcome of the probiotic.

Surprisingly, only three of these human studies examined whether the microbiome composition was impacted following probiotic use [100,102,103]. One found minimal effects on the composition as a whole, but did observe an increase in the *Lactobacillus* genera that was being supplemented [103]. A second also observed an increase in the *Bifidobacterium* genera used in the probiotic supplementation, with additional subtle compositional shifts including an enrichment in fiber fermentative *Ruminococcaceae* and *Lachnospira*, and decreases in *Lactobacillus fermentum* and *Klebsiella oxytoca* [100]. This is notable since these are PD-associated taxa that are decreased or enriched respectively, and in this case probiotic intervention shifts these taxa to less PD-like state. The third study did not observe enrichment of the taxa that were administered, but did observe an increase *Christensenella Marseille-P2437* but decreased levels of *Eubacterium oxidoreducens, Eubacterium hallii*, and *s_Odoribacter_sp. _N54.MGS-14* [102]. Microbiome compositional evaluations are currently lacking in published probiotic-PD clinical trials. Ongoing trials now list microbiome evaluations as an outcome measure, in some cases, even as the primary outcome (NCT05146921). Such assessments are necessary to understand whether the administered taxa need to be enriched to have benefit, or transiently interact with the microbiome. Further, secondary effects on the entire community structure may be important to provide benefit, even if a single species is being administered.

All of the clinical trials currently published on probiotic therapy for PD selected probiotic strains that have been used in humans to treat ailments in other disease states, rather than identifying specific species that are depleted in the PD-associated microbiome. However, there has been some recent interest in treating PD with probiotic species that are more closely associated with human PD, or which had more unexpected findings in experimental models. One such study (NCT05832775) aimed to treat PD with *Megasphaera massiliensis* (MRx0029) and *Parabacteroides distasonis* (MRx0005). Neither of which have a consistent association with PD [15]. In fact, *P. distasonis* has been observed to be increased in one large study [32], although it has been observed to improve inflammatory outcomes in arthritis and colitis [112,113]. Another ongoing clinical trial (NCT06487975) is testing the efficacy of the probiotic *Bacillus subtilis*, based on preclinical data in *C. elegans* [66] that found that *B. subtilis* inhibits α-synuclein aggregation and can even clear preformed aggregates. Identifying the contributions of specific bacterial species that are consistently depleted in PD, would inform which species may be most prudent to further test. Additionally, given complex microbe-microbe interactions within the intestinal environment, including cross-feeding and niche competition, identifying keystone species with a capacity to restore the community as a whole to a non-PD state would be a critical next step in the informed development of probiotic treatment for PD.

## Dietary and Prebiotic Treatments

Enrichment or exposure to specific microbes in probiotic approaches is just one precise methodology to potentially improve microbiome-dependent signals. Administration of dietary fiber (*i.e.* prebiotics) and other diet-based interventions, provides substrates for microbiome-mediated metabolism. These approaches can also allow for certain microbes to be enriched within the community, due to preferences for energy sources, as well as impact the functional output of the microbiome, even if limited in its compositional shifts. While some studies discussed in prior sections combined probiotic and prebiotic fiber supplementation [98,101], a few clinical and preclinical studies have evaluated the efficacy of treating aspects of PD using prebiotics and dietary supplementation alone.

One clinical trial found that consumption of 10 ​g of resistant starch as a prebiotic per day over an 8-week trial period significantly improved depression and non-motor symptoms of PD [114]. Despite a lack of significant changes to the composition of the gut microbiome, fecal levels of the anti-inflammatory and multi-functional metabolite butyrate were also increased, and fecal levels of the inflammatory calprotectin were decreased in the treatment group. A more recent study used a well-defined prebiotic fiber bar- a combination of resistant starch, rice bran, resistant maltodextrin, and inulin [115]. Here, the study team identified that this fiber intervention substantially altered the gut microbiome and improved inflammatory biomarkers of PD [115]. Specifically, prebiotic intervention increased the abundance of short-chain fatty acid (SCFA)-producing bacteria and reduced the abundance of potentially pathogenic bacterial taxa (*e.g. Enterobacteraceae*) in stool. Similar to prior findings [114], SCFA levels, including butyrate, were increased and fecal calprotectin was decreased, indicating a shift toward a beneficial anti-inflammatory environment within the intestinal lumen. Importantly, this study also identified a significant reduction in circulating levels of neurofilament light chain, a common marker for neurodegeneration, suggesting that fiber intervention was capable of slowing neuronal death. These findings in human cohorts are further validated in animal models. For instance, administration of prebiotic fibers improved motor and non-motor functions in the rotenone mouse model of PD [116,117]. In addition, feeding alpha-synuclein overexpressing mice (Thy1-hSNCA) a prebiotic-enriched diet containing wheat bran and resistant maltodextrose reduced α-synuclein aggregation and improved motor outcomes [118]. This process was associated with a dampened microglia inflammatory response, implicating a role for anti-inflammatory SCFA signaling to mediate beneficial gut-to-brain activities.

A common finding in prebiotic studies across both human and animal models (both within and outside the context of PD), is an increase in SCFAs, most notably butyrate. This molecule is well-established to be diminished in PD [119,120], and decreased SCFA levels are also associated with other inflammatory and neuro-inflammatory disorders. Butyrate is a potent anti-inflammatory compound that works both through immune-modulating FFARs and by inhibition of histone deacetylase activity and has been shown to improve symptoms in various diseases [121]. Building upon this, a handful of post-biotic studies have been conducted to test the downstream microbiome-mediated metabolites of butyrate, butyrate prodrugs, or butyrate-enriched foods. These have been observed to lead to improvements of PD symptoms in humans [122,123], rats [124,125], mice [126], and *Drosophila* sp [127]. Importantly, different types of prebiotics lead to different profiles of SCFAs, the production of which also depends on microbiome composition. For this reason, fiber combinations have been selected for preclinical [118] and clinical [115] PD studies based on their fermentation profiles by PD-derived microbiomes. A number of prebiotic and dietary interventions are currently being investigated in clinical trials as therapeutics for PD [128].

Other than specific fiber consumption, a breadth of epidemiological evidence supports the notion that the dietary consumption of tea and coffee is protective against PD risk [[129], [130], [131], [132], [133]]. One particular study, in a cohort of near 30k individuals, found that daily coffee or tea consumption decreased the risk of PD by half [131]. One of the major components of coffee and tea that can have direct effects on the microbiome are the polyphenols. Polyphenols (*e.g*. catechins, quercetin, fisetin, anthocyanin, curcumin, isoorientin) are naturally occurring plant-derived metabolites with demonstrated neuroprotective actions both *in vitro* and *in vivo* through regulation of immune, mitochondrial, proteostasis, and reactive oxygen pathways [134]. Notably, dietary polyphenols modulate both the composition of the gut microbiome and beneficially impact the intestinal environment [[135], [136], [137], [138]]. High consumption of one class of polyphenols, flavonoids, was seen to result in a 40 ​% reduction in PD risk compared to low consumption counterparts [139].

Studies in mice, rats and *Drosophila* sp. have shown that one polyphenol in particular, epigallocatechin gallate (EGCG), has promising ability to reduce amyloid protein accumulation, for instance amyloid β and alpha-synuclein. EGCG treated amyloid-transgenic mice displayed improved performance on cognitive assessments [140]. Similarly, EGCG also showed a stark prevention of MPTP-induced neurodegeneration in mice [141,142]. The anti-amyloidogenic properties of EGCG also limits the formation of bacterial amyloids, such as *Enterobacteriaceae*-derived curli proteins [143]. Since curli has the ability to trigger alpha-synuclein pathologies *in vitro* and in mouse, rat, and worm models of PD pathologies [52,[58], [59], [60]], it is possible that EGCG prevents such pathogenic interactions between bacterial and host amyloids, like synuclein. Indeed, a clinical trial is ongoing investigating the safety and efficacy of EGCG (in the form of green tea extract) for PD (NCT00461942). Since polyphenols are not readily absorbed into circulation [134], benefits of these molecules are likely due to local effects within the GI tract and/or on the functional interactions of the microbiome.

These prebiotic approaches to modulate the gut microbiome in PD provide a rapid and safe alternative to probiotic or FMT therapies, that may also provide benefit by specifically influencing the metabolic output of an existing microbiome and potentiating long-term compositional shifts through nutrient selection. However, prebiotic interventions necessitate the presence of organisms that can metabolize the given dietary input. Some microbiomes of individuals with PD, for instance, have an exceptionally low abundance of fiber-fermenting organisms. This may make prebiotic interventions less effective in this population and highlights a need to better stratify individuals with PD based on discrete taxa with known contributions.

## Antibiotic Treatments and Other Emerging Methodologies for Therapeutic Microbiome Manipulation

Beyond the more conventional microbiome approaches listed above, a number of emerging therapies utilizing novel means to target the microbiome are currently being investigated. Approaches of this nature currently in clinical trials include the use of antibiotics and microbiome-directed small molecules in the treatment of PD. Other next-generation methodologies to manipulate the microbiome are still being evaluated in model systems, or have yet to be assessed in PD - including bacteriophage therapy and CRISPR-mediated targeting. These emerging and potential future therapies are predicted to have the capacity to selectively shift the microbiome to restore or limit microbes that are thought to contribute to PD pathologies.

Of these therapies, the most well characterized thus far are antibiotics. Antibiotics defined here are any compound capable of inhibiting the growth of, or directly killing, microorganisms. Rifampin, an ansamycin antibiotic regularly used to treat tuberculosis, and doxycycline, a tetracycline, have shown neuroprotective promise in both *in vitro* and *in vivo* studies with anti-apoptotic, anti-inflammatory, and anti-oxidative properties [[144], [145], [146], [147]]. *In vitro* direct exposure of purified alpha-synuclein protein to either antibiotic resulted in a reduction of alpha-synuclein oligomers forming fibrils (the pathological form of the protein found in PD) and even a disaggregation of alpha-synuclein fibrils into oligomers [148,149]. Similarly, in rat neuroendocrine cells exposed to MPP+, rifampin decreased alpha-synuclein accumulation and dose-dependently increased cell viability [150]. Cetriaxone, a β-lactam antibiotic, has also shown neuroprotective properties, potentially partially through synuclein related mechanisms [151,152]. Thus, even in the absence of a microbiome, antibiotics may have some pharmacological features that are beneficial against hallmark PD pathology.

The cephalosporin antibiotic, ceftriaxone, was able to protect against MPTP induced neurodegeneration in rats. Neuroprotection in these cases was attributed to a marked reduction in oxidative stress, inflammation, and excitotoxicity in ceftriaxone treated mice [35,37]. While ceftriaxone is known to impact the gut microbiome [153], these particular studies examining neuroprotection to MPTP did not directly assess the microbiome composition. To our knowledge, at least three clinical trials evaluating antibiotics for PD are underway. A Phase 2 clinical trial, which began in 2019, is testing whether cognitive function can be improved in PD patients given ceftriaxone (NCT03413384). Erythromycin is being evaluated to improve levodopa absorption and improve motor outcomes (NCT02005029). A third is evaluating rifaximin, which is gut-restricted, to investigate its ability to reduce levodopa metabolizing bacteria and improve pharmacological efficacy (NCT03575195). These human studies, once complete, will greatly inform the continued study of common antibiotic interventions to improve symptoms in PD. Beyond single antibiotics, broad-spectrum cocktails have also been assessed for their ability to prevent microbial dysbiosis and limit PD pathologies. In one experimental study [38], the administration of a cocktail containing ampicillin, neomycin, and metronidazole for 14 days prior to MPTP exposure, prevented neurodegeneration in mice. In the Thy1-hSNCA alpha-synuclein overexpression mouse model, a cocktail of ampicillin, neomycin, vancomycin, gentamycin, and erythromycin limited pathologies and motor deficits [33], although in the A53T mouse model, this same cocktail had no significant effects on motor outcomes [34]. It should be noted that in these animal studies, the use of chronic, broad-spectrum antibiotics is used to deplete the microbiome, rather than mirror the known microbiome perturbations to humans following a therapeutic course of antibiotic treatment.

While antibiotic use decreases microbial abundance and diversity [154], their actions are not wholly selective, and they may decrease the abundance of potentially beneficial species [155] and/or select for antibiotic-resistance genes within the microbiome [156]. In addition, some epidemiological studies report a positive association with use of certain antibiotics (macrolides, lincosamides, tetracyclines, and penicillin) and PD diagnosis in European cohorts [157,158]. The reason for this association is not clear, particularly given that the broad associations currently described encompass antibiotics with varied mechanism of action across both Gram positive and negative species. It may be that more frequent antibiotic use significantly disrupts the gut microbiome and predisposes an individual to a detrimental inflammatory environment that increases risk of PD. Alternatively, these findings could also be suggestive that those with more frequent/more severe microbial infections that necessitate antibiotic use, are at greater risk for PD.

Next-generation techniques to modify the microbiome include the use of bacteriophages and CRISPR technologies. Both of these have shown promise experimentally in altering microbiome composition and/or introducing novel genetics to specific microbes within a complex community [[159], [160], [161]]. Bacteriophages are an understudied contributor to disease-associated microbiomes in general and play key roles in ecological dynamics and gene transfer within the community. The PD-associated microbiome, given the large difference in its bacterial composition, also has significant differences in the abundances of bacteriophages [[162], [163], [164]]. Bacteriophages are an attractive tool due to their high specificity to a bacterial host, and limited interactions with the mammalian host immune systems. The use of selective phages to deplete pathogens from both homogenous and heterogenous infections has been observed in a number of systems, including the ESKAPE pathogens (*Enterococcus faecium*, *Staphylococcus aureus*, *Klebsiella pneumoniae*, *Acinetobacter baumannii*, *Pseudomonas aeruginosa* and *Enterobacter* spp.) [[165], [166], [167]] and recently treatment-resistant Mycobacteria [168]. Further understanding of the species specificity of those bacteriophages within the gut microbiome, as well as the determinants of such specificity are greatly needed. Another highly selective technology, CRISPR/Cas9, is an RNA-guided endonuclease with relatively high specificity to introduce DNA breaks at desired sites [169]. In the context of the microbiome, bacteriophage-delivered CRISPR has recently been used to deplete specific bacteria from within a community and alter the gut microbiome in mice [159]. In these studies, CRISPR machinery, delivered with an *E. coli* specific phage in mice showed the capability to selectively remove bacterial strains [159,160]. However, with both these technologies it is unknown how selective removal (or pressure) of one species may impact the rest of the community. In the context of PD, it is currently unknown whether specifically removing one or two species from the PD-associated gut microbiome would provide benefit, even in animal models.

CRISPR has also recently been used to alter the genetics of specific species within the gut microbiome to modify their functionality, without altering their abundance or overall community structure [170]. New technology involving selective microbial gene editing might provide a novel microbiome focused approach to treatment of PD, where not only can bacterial species that bloom in PD be eliminated, but beneficial genetic processes can be introduced to other indigenous species. With tangible examples from experimental contributions known today, this could include targeting those bacterial enzymes responsible for levodopa metabolism to improve existing therapeutics. Or, it may be considered to selectively remove bacterial amyloids from a complex population. These next-generation methods have only been tested in proof-of-concept animal studies. Their ability to actively modulate a microbiome in humans or microbiome-dependent physiological responses (let alone, PD-related pathologies) have not yet been explored. Nonetheless, they all represent exciting new tools for therapeutic development.

## Looking ahead at the Microbiome-therapeutic Landscape for PD

The microbiome has emerged over the past decade as a potential contributor to PD pathologies. With its ease of manipulation, microbiome-directed therapeutics have begun to show significant benefits across a number of animal models. Recent and ongoing clinical trials have demonstrated exciting promise; however, results are mixed across these studies.

Continued use of experimental gnotobiotic models will allow the precise identification of specific contributions of PD-associated microbes to pathologies. While we understand that some microbes can contribute to relevant pathology, understanding roles for individual microbes will guide development of selective therapeutics. For instance, designing selective antibiotics or bacteriophages to specifically target organisms with demonstrated pathological contributions, or enriching significantly depleted microbes with experimentally-demonstrated beneficial actions is preferable to relying on more outdated views of “probiotics” that may use organisms with less relevance to PD pathogenesis. In line with this, the term “Live Bacteriotherapeutic Products” (LBPs) has begun to be used to describe treatments with targeted beneficial organisms, in contrast to the use of broad probiotic organisms which may not necessarily be linked to a given disease etiology. While microbiome-dependent outcomes in PD animal models strongly demonstrate a role for the microbiome in these systems, knowledge of the precise mechanisms of action is much more limited. A better understanding of the cellular and molecular pathways acted on by the microbiome to promote or limit PD pathologies will identify core components of disease that may be targeted therapeutically, even independently of the microbiome.

PD does not manifest as a homogenous syndrome, and individuals living with PD experience varying repertoires of motor and non-motor symptoms. Some of these may represent differing etiologies that underlie the shared loss of vulnerable neurons. A refined view of predicted PD subtypes, for instance the brain-first vs. body-first phenotypes, and supported pathological etiologies, may reveal populations that are more likely to respond to microbiome-directed therapeutics. Similarly, the human microbiome is variable even within those living with PD, and subtypes of microbiomes based on characteristic taxa exist within the population. Clarifying whether these microbiome subtypes associate with particular aspects of disease, in conjunction with their evaluation in experimental models may reveal microbiome compositions that contribute more strongly than others- warranting their preferential inclusion in clinical studies [171].

Continued study of the *mechanistic* contributions in experimental models, combined with rigorous analysis of the human population with PD using deep phenotyping (PET imaging, seed amplification assays), and longitudinal microbiome assessments of at-risk individuals, will greatly enhance our knowledge of when and how the microbiome contributes to PD and which individuals may most benefit from a microbiome-directed therapeutic. Overall, these next generations of microbiome-based therapeutics may hold promise for this recalcitrant disease.

## Author Contributions

All authors wrote and revised this review.

## Declaration of competing interest

TRS is an author of published patents (institutionally-assigned) surrounding microbiome therapeutics for Parkinson's disease.
